# The value of a causal loop diagram in exploring the complex interplay of factors that influence health promotion in a multisectoral health system in Australia

**DOI:** 10.1186/s12961-018-0394-x

**Published:** 2018-12-29

**Authors:** Lori Baugh Littlejohns, Fran Baum, Angela Lawless, Toby Freeman

**Affiliations:** 1 Menzies Centre for Health Policy, The Australian Prevention Partnership Centre, D17 Charles Perkins Centre, University of Sydney, Sydney, NSW 2006 Australia; 2 Southgate Institute for Health, Society and Equity, Flinders University, South Australia Bedford Park, Australia; 3 Discipline of Speech Pathology, Flinders University, South Australia Bedford Park, Australia; 4 Research Affiliate, University of Sydney, Sydney, Australia

**Keywords:** Health promotion, System building blocks, Complex systems thinking, Causal loop diagram

## Abstract

**Background:**

Despite calls for the application of complex systems science in empirical studies of health promotion, there are very few examples. The aim of this paper was to use a complex systems approach to examine the key factors that influenced health promotion (HP) policy and practice in a multisectoral health system in Australia.

**Methods:**

Within a qualitative case study, a schema was developed that incorporated HP goals, actions and strategies with WHO building blocks (leadership and governance, financing, workforce, services and information). The case was a multisectoral health system bounded in terms of geographical and governance structures and a history of support for HP. A detailed analysis of 20 state government strategic documents and interviews with 53 stakeholders from multiple sectors were completed. Based upon key findings and dominants themes, causal pathways and feedback loops were established. Finally, a causal loop diagram was created to visualise the complex array of feedback loops in the multisectoral health system that influenced HP policy and practice.

**Results:**

The complexity of the multisectoral health system was clearly illustrated by the numerous feedback mechanisms that influenced HP policy and practice. The majority of feedback mechanisms in the causal loop diagram were vicious cycles that inhibited HP policy and practice, which need to be disrupted or changed for HP to thrive. There were some virtuous cycles that facilitated HP, which could be amplified to strengthen HP policy and practice. Leadership and governance at federal–state–local government levels figured prominently and this building block was interdependently linked to all others.

**Conclusion:**

Creating a causal loop diagram enabled visualisation of the emergent properties of the case health system. It also highlighted specific leverage points at which HP policy and practice can be improved. This paper demonstrates the critical importance of leveraging leadership and governance for HP and adds urgency to the need for increased and strong advocacy efforts targeting all levels of government in multisectoral health systems.

**Electronic supplementary material:**

The online version of this article (10.1186/s12961-018-0394-x) contains supplementary material, which is available to authorized users.

## Background

The application of complex systems science to health promotion (HP) has much promise [1]. There are, however, few published empirical studies that discuss its application in order to study HP policy and practice and demonstrate its practical value. This paper reports on the application of a complex systems approach to study the key factors influencing HP policy and practice in an Australian multisectoral health system. First, an explanation of how HP and a complex health system are conceptualised followed by the gaps identified in the literature are provided.

### Health promotion

The WHO definition of HP is “*the process of enabling people to increase control over, and to improve their health. It moves beyond a focus on individual behaviour to consider a wide range of social and environmental interventions*” [2]. This definition points to the importance of multilevel (individual through to societal) and multisectoral (many sectors, including health) action on the social, economic and environmental determinants of health as central to the desired HP policy and practice. Evidence indicates that these structural drivers in society are pivotal determinants of health inequities [3–6].

This paper takes the goal of HP as promoting overall population health and reducing health inequities, that is, the preventable and unfair distribution of the determinants of health [4]. The conceptualisation of HP used in this paper is based on WHO’s Ottawa Charter for Health Promotion [2]. Reorienting health services towards HP [2, 6], ensuring community participation in identifying and addressing priority determinants of health [2, 7], and developing partnerships and intersectoral collaboration to take coordinated action [8] can be regarded as the fundamental processes or actions through which HP strategies need to be planned, implemented and evaluated (Table 1). Developing personal skills, creating supportive environments and building healthy public policies [2] represent three strategies to take action to address the goal of HP (Table 1).

**Table 1:** Conceptualisation of health promotion (HP)

HP Goal (why) Promote population health and reduce health inequities through action on the broad social, structural, economic, political, environmental and behavioural determinants of health	
HP Actions (how) Ensure community participation in HP Develop partnerships and intersectoral collaboration for HP Reorient health services toward HP	HP Strategies (what) Develop personal skills Create supportive environments Build healthy public policy

Although international documents have long recommended the actions and strategies described above, there remain significant challenges for multisectoral health systems (further described below) to adopt policies and practice that are focused on reducing health inequities [3, 4, 9, 10].

### Complex health systems

**Fig. 1:**
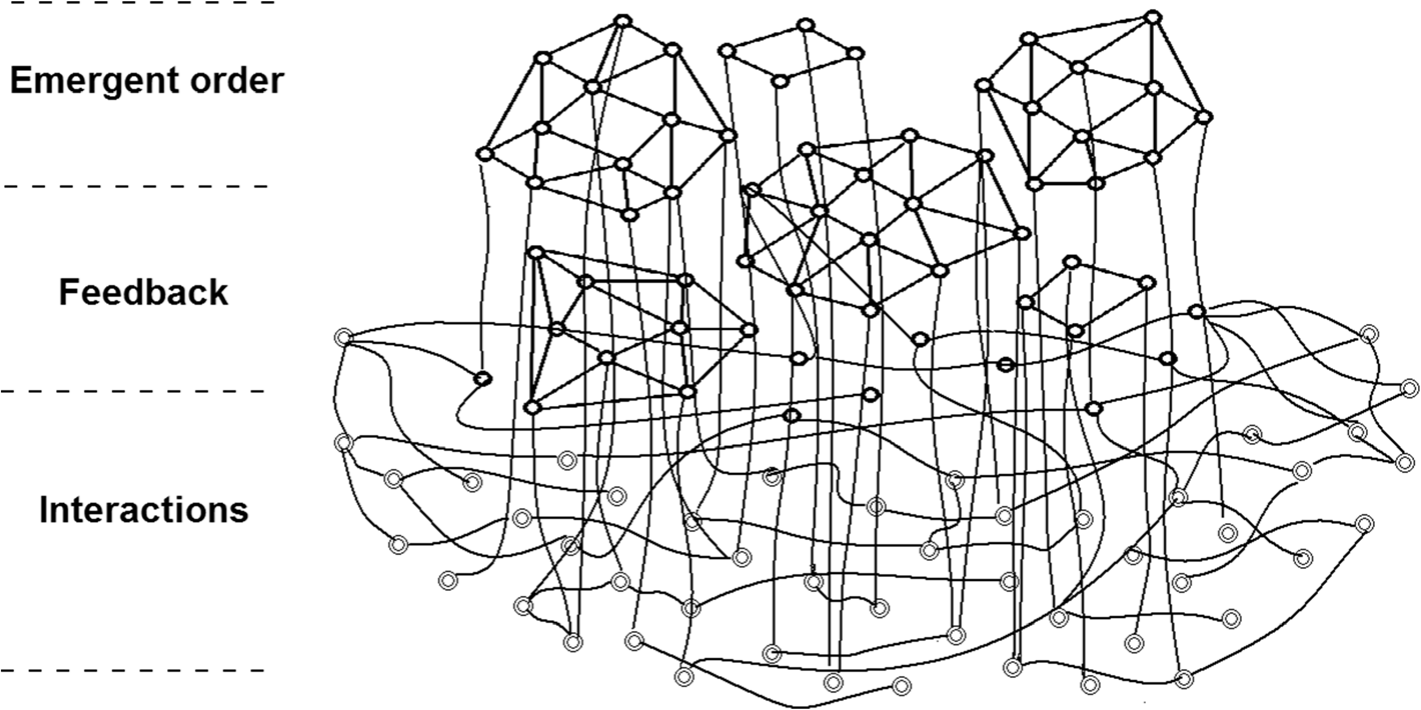
Three characteristics of complex systems [32] (used with permission from A. Strauss & Associates; http://maverickandboutique.com)

HP is challenging, not only in terms of the range and interrelationships among determinants of health, but also the complex systems that shape HP policy and practice [11]. Health systems can be described in terms of the broad and numerous social systems that influence health and well-being as well as clinical healthcare services [12]. Multisectoral health systems are complex, primarily because of interactions, feedback and emergent order within systems [13–17]. Figure 1 illustrates these characteristics and their relationship to one another.

#### Interactions

Complex systems have numerous nested and heterogeneous system elements that exhibit considerable variation, with each element being a system in their own right [18–20]. Health systems are complex because they are comprised of multiple entities, organisations, agencies and sectors at local, regional, state, national and international levels, all of which vary in terms of their structure, function and interests. Each element has a unique relationship to and influence on the whole health system [21]. Key to understanding multisectoral health systems are the interactions among elements that influence the overall health system [13, 18, 22].

#### Feedback

Complex systems are dynamic because of their continuous ability to change, adapt and reorganise in response to their environment [23]. Self-organisation is a concept used to describe the adaptation of systems to their environment and to study how systems organise, change and/or innovate [24–26]. Feedback loops are the interconnections that illustrate self-organisation in complex systems [25, 27]. The behaviour of complex systems is in large part the accumulative effect of positive (reinforcing or self-enhancing) and negative (balancing or goal seeking) feedback mechanisms [24, 28]. ‘Virtuous’ and ‘vicious’ are descriptors of feedback loops that are going in favourable or unfavourable directions.

#### Emergent order

Interactions and feedback mechanisms produce emergent order or properties of the whole system [29]. Emergent properties therefore cannot be inferred by the study of individual system elements or variables but rather through the study of relationships in the whole system [26]. Other factors that influence emergence include the history and context of the system [30, 31]. Emergent order in health systems was described by Jayasinghe as follows: “*patterns of population health outcomes are an emergent property of the system. They arise from a web of causations that result from interactions among dynamic sets of interconnected systems*” ([33], p. 5).

### Health system building blocks

One way to study health promotion in complex health systems is through the lens of WHO’s health system building block framework [34]. The framework describes the key capacities or building blocks needed for effective functioning, and these provide a way to study the complexity of health systems in terms of the interactions and feedback mechanisms among building blocks and the resultant emergent order. Harnessing the synergies created between interacting building blocks is considered instrumental to achieving health system goals or a desirable emergent order [35]. The adaptation of the framework to the study of a multisectoral health system for HP is described below.

### Gaps in the literature

Despite the potential of using a complex systems approach that incorporates health system building blocks to study HP in multisectoral health systems, this appears not to have been done previously. Further to this, few studies focus on the interactions and feedback mechanisms that influence the emergent order in health systems with respect to HP policy and practice. The study of interactions and feedback could be very enlightening as Allender et al. [36] showed in terms of the causes of obesity in Australia. In a similar vein, the Foresight Centre [37] in the United Kingdom also illustrated interactions, feedback and emergent order with respect to causes or determinants of obesity within systems. In a Canadian HP policy study, Alvaro et al. [38] found a “*lopsided*” emphasis on individual lifestyle and behavioural approaches. They discussed positive or reinforcing feedback loops characterised as vicious cycles because they maintain the focus on HP strategies targeting individuals as opposed to a balance of strategies that also address societal or structural determinants of health. Building on these examples, a study of the interactions and feedback mechanisms among system elements and building blocks and the emergent order created in a multisectoral health system for HP appeared a promising way forward. This paper reports on research assessing the feedback mechanisms that appear to influence HP policy and practice in a multisectoral health system in Australia.

## Methods

The first part of this section describes a case study approach and indicates the sectors, system elements and levels that bound the case and, thus, the multisectoral health system. Boundaries are the borders between complex systems and their environments and these are often “*fuzzy*” [39]. This ‘fuzziness’ applies to complex health systems and, by drawing boundaries of a multisectoral health system (i.e. delineating system elements, stakeholders and variables), it is possible to study feedback mechanisms [18]. Following this, data collection and analysis methods are explained, including document review, interviews, coding and how feedback mechanisms were identified.

### Case study

This research was a single instrumental case study [40] and used qualitative methods. Luck et al. describe a case study as “*a detailed, intensive study of a particular contextual and bounded phenomena that is undertaken in real life situations*” ([41], p. 104). The case was a multisectoral health system in a region of South Australia (SA). The region is not identified at the request of stakeholders who were interviewed.

The multisectoral health system that formed the case study was selected based on the following attributes: (1) it was bounded in terms of geography and institutional governance structures of a Local Health Network and had co-terminus boundaries with four local governments (see Table 2 below for descriptions); (2) there were numerous and diverse sectors and system elements with roles in HP; and (3) there was a history of support for and action on HP, including intersectoral collaboration among sectors and subsystems [44]. At the time of the research (2013), the multisectoral health system was shaped by numerous federal, state, regional and local entities (i.e. sectors and system elements) and a range of governance structures. Table 2 provides a brief description of relevant sectors and system elements that were in place in the multisectoral health system.

**Table 2:** Description of sectors and system elements in the multisectoral health system

Sector	System elements	Description
Federal government: health sector	The Department of Health of the Australian Government	*Federal:* Department of Health portfolios included population health (health promotion; HP), pharmaceutical services, medical and dental services, acute care, primary healthcare, private health, infrastructure, regulation, safety, quality, workforce capacity, biosecurity, and sport and recreation
	Medicare Locals (changed to Primary Health Networks in July 2015) were established through the Department of Health, Australian Government and had a large geographical area that included geographical boundaries of the Local Health Network (state managed regional entities as described below)	*Regional:* Medicare Locals were federally funded regional institutions responsible for priorities and reporting with respect to the coordination of primary healthcare services, addressing local healthcare priorities, supporting health professionals, and improving access to primary care
State government: health sector	SA Health (Government of South Australia health department)	*State and regional:* SA Health was responsible for public hospitals (with a joint agreement with the Australian Government), health service delivery, public health (environmental health, epidemiology, communicable disease control, HP), pathology services, drug and alcohol services, dental services, GP Plus health centres, emergency and ambulance, and organ donation
	Local Health Network (state-managed regional primary healthcare services)	*State and regional:* Under the direction of SA Health, Local Health Networks managed acute, sub-acute and mental health services delivered in public hospitals and GP Plus Centres. Networks were defined geographically (e.g. Southern Adelaide, Northern Adelaide) or functionally (Women’s and Children’s Health)
State government: other sectors	State government departments	*State and regional:* Services and resources in areas such as education, family support, sport, recreation, and transportation
Local governments	Local Councils had co-terminus boundaries with the Local Health Network	*Local:* Local Councils were the legislated public health authority for their geographical area with responsibilities to preserve, protect and promote health, ensure adequate sanitation measures are in place, identify public health risks, respond to impacts upon public health, prepare public health plans, and provide immunisation services
Non-governmental organisations (NGOs)	Three types of NGOs were identified as elements of the system: professional associations, health service delivery organisations, and intersectoral networks	*State:* professionals associations *State and regional*: health service delivery organisations (e.g. sexual health) *Regional and local:* intersectoral networks of regional and community service delivery organisations

### Data collection and analysis

Table 3 provides an overview of data collection and analysis methods. To summarise, document analysis was used to first assess the extent to which the policy context – as formally articulated in policy and related strategic documents – supported the goal, actions and strategies conceptualised for HP and health system building blocks for HP. Interviews were then conducted with stakeholders in leadership roles in HP (Tables 2 and 3) to explore their perspectives of and experiences in the HP policy and practice environment. A semi-structured interview guide was used to ask questions in the following areas: details of individual and organisational roles in HP, descriptions of and changes in the HP policy and practice environment, and perspectives concerning the key factors that influence HP policy and practice. Interviews provided contextual information and explored the implementation of policy intentions.

**Table 3:** Data collection and analysis methods for document review and interviews

Method	Description
Document review	Analysis of a purposeful sample [42] of 20 SA government policies and strategic documents from 2003 to 2013 that were relevant to health promotion (HP) policy and practice in SA was completed All documents were imported into QSR NVivo 10, and coded and analysed from November 2012 to May 2013 Directed content analysis methods were used to establish key findings [43]
Stakeholder interviews	Interviews were conducted with a purposeful sample of 53 stakeholders in multiple sectors with leadership roles (e.g. Mayor, Director, President, Manager, Coordinator) in HP working within the multisectoral health system The sample included stakeholders from the following entities making up the system elements: ▪ Four local governments and the state local government association (*n* = 16) ▪ Three health sector entities (*n* = 16): SA Health [the state health department] (*n* = 5); one Local Health Network [state managed regional health services] (*n* = 6); and one Medicare Local [federally funded regional managed primary healthcare organisation now called Primary Health Network] (*n* = 5) ▪ Three non-government sector entities (*n* = 18): intersectoral networks (*n* = 6), professional associations (*n* = 8), and health service agencies (*n* = 4) ▪ Three other state government departments (*n* = 3) Face-to-face and telephone interviews were conducted (all individual except two group interviews: one with SA Health and the other with Local Health Network interviewees) All interviews were conducted from May 2013 to December 2013, transcribed, and imported into QSR NVivo 10 for coding and analysis; directed content analysis methods were used to establish key findings [43]

Table 4 provides the unique coding schema for document review and interview data. Using NVivo software, documents and interview transcriptions were coded according to their reference to the HP goal, actions, and strategies and health system building blocks. Definitions of health system building blocks [34] were adapted to better reflect the capacities needed for HP in multisectoral health systems. Further, ‘medicines and technologies’ was not included in the schema as it relates mostly to clinical healthcare in the health sector as opposed to multisectoral health systems for HP.

**Table 4:** Coding schema

Code	Component	Guiding definition
Health Promotion (HP) Goal	Promote population health and reduce health inequities	HP needs to focus “*on achieving equity in health reducing differences in current health status and ensuring equal opportunities and resources to enable all people to achieve their fullest health potential*” [2] and reducing health inequities through action on the social determinants of health as a clear goal [4]
HP Action	Ensure community participation	“*Health promotion works through concrete and effective community action in setting priorities, making decisions, planning strategies and implementing then to achieve better health*” [2]
HP Action	Develop partnerships and intersectoral collaboration	“*Health promotion demands coordinated action by all concerned: by governments, by health and other social and economic sectors, by nongovernmental and voluntary organizations, by local authorities, by industry and by the media*” [2]
HP Action	Reorient health services toward HP	“*The role of the health sector must move increasingly in a health promotion direction, beyond its responsibility for providing clinical and curative services*” [2]
HP Strategies building block	HP services (practice)	HP requires the implementation of multiple strategies at multiple levels including: Develop personal skills “*Health promotion supports personal and social development through providing information, education for health, and enhancing life skills*” [2] Create supportive environments “*… refers to both the physical and the social aspects of our surroundings … determines access to resources for living, and opportunities for empowerment … has many dimensions: physical, social, spiritual, economic and political*” [7] Build healthy public policy “*Healthy public policy is characterized by an explicit concern for health and equity in all areas of policy and by an accountability for health impact*” [45]
Building block	Leadership and governance	Leadership and governance for HP ensures “*strategic policy frameworks exist and are combined with effective oversight, coalition building, regulation, attention to system-design and accountability*” [34]; this was adapted to include governance for health (HP action of developing partnerships and intersectoral collaboration) and health governance (HP action of reorienting health services) [46]
Building block	Financing	The provision of adequate funding for all system building blocks for HP in order to achieve the goal of reducing health inequities (adapted definition)
Building block	Workforce	The presence of an adequate, efficient and responsive workforce with sufficient numbers of trained people (adapted definition)
Building block	Information	The production, analysis and dissemination of reliable and timely information on health determinants, health status and health system performance [34]; this was adapted to include HP research and evaluation

Following coding and analysis, a summary of key findings was completed for both document analysis and interview data. A detailed discussion of the document review analysis as well as results can be found elsewhere [47]. Based on key findings, a complex system lens was applied to identify interactions, feedback and emergent properties in the multisectoral health system with respect to HP policy and practice. Kim and Andersen’s [48] process was adapted to link key findings from document review and interviews to feedback mechanisms through the identification of dominant themes. This involved five steps, as follows:

1. When a key finding was found in both data sets, it was labelled a dominant theme (Table 5).
2. Causal links were then identified among dominant themes and key findings. Several criteria described by Davidson [49] for inferring causality were used including temporal precedence (i.e. establishing A before B), constant conjunction (i.e. when A, always B), and contiguity of influence (i.e. plausible mechanisms for linking A and B). This process was intensely iterative and ended only when each causal link was clearly substantiated.
3. Following this, causal links were translated into words-and-arrows diagrams with each representing an interaction.
4. When a causal link demonstrated a reciprocal relationship, a feedback loop was created. Each feedback loop was assessed in terms of its polarity (positive polarity signifying a reinforcing relationship and negative polarity signifying a balancing relationship) thus establishing whether the loop was a facilitating or inhibiting factor for HP policy and practice [50].
5. All feedback loops were then assembled into a causal loop diagram to create a visual model [51]. Vensim PLE software was used to create word-and-arrow diagrams, feedback loops and the causal loop diagram. In the interest of providing a more reader-friendly diagram, facilitating (happy face) or inhibiting (sad face) influences on HP policy and practice in the case health system were used (i.e. the polarity of each feedback loop is not labelled).

**Table 5:** Key findings regarding factors that influenced health promotion (HP) policy and practice

Key findings	Document review	Stakeholder interviews
Lack of strong support for or discussion of reducing health inequities	✓	✓
Lack of support for community participation	✓	✓
Lack of clear federal-state-local government roles, governance structures and policy directions	✓	✓
Cuts to/lack of HP financing	✓	✓
Cuts to/the need for HP workforce capacity	✓	✓
Cuts to/limited HP service	✓	✓
Lack of information/evidence of HP effectiveness	✓	✓
Negative impact of state economic circumstances/budgetary constraints	✓	✓
Calls for/focus on whole-of-government approaches	✓	✓
Potential negative impact of state leadership changes to HP		✓
Negative impact of HP discourse regarding past financing and services in health sector		✓
Dominance of the biomedical model		✓
Demoralisation of HP workforce		✓
Fear of cost shifting from state to local governments		✓
Fragmented system elements		✓
Need for a strategic framework		✓
Support for monitoring and reporting on population health	✓	

## Results

First, an overview of the HP policy and practice context followed by key findings from the document analysis and interviews are presented. The next section interweaves reporting on dominant themes and the feedback mechanisms identified. Finally, the causal loop diagram portraying all feedback mechanisms in play in the case study health system with respect to HP policy and practice is described.

### Overview of HP policy and practice context

The policy context changed from strong advocacy for HP in 2003 to its near abandonment in 2013. From 2003 to 2011 there was considerable support for HP but this support diminished significantly in 2013 following the Review of Non-hospital Based Services [52] (hereafter called the Review) and SA Health’s Response [53]. The government’s response to the Review resulted in substantial cuts to HP financing, workforce and services, which are essential health systems building blocks. Documents identify that cuts were made because of (1) the poor state economic environment, rising healthcare costs and the need for budgetary constraints, (2) unclear federal-state roles, governance structures and policy directions, and (3) the lack of evidence regarding HP effectiveness [52]. More positively, the SA Public Health Act provided a foundation for partnership, intersectoral collaboration and whole-of-government approaches to HP. All interviewees, except those from the state health department (5 of 53), described the HP policy and practice environment in very negative terms because of the heavy cuts to HP proposed by the Review and accepted in SA Health’s Response. Several participants said that HP was now a “*dirty word*” (NGO/Health Service/Professional Association, Local Government). Other descriptors included “*big void*”, “*devalued*”, “*devastating*”, “*dire*”, “*expendable*”, “*obliteration*”, and “*toxic*”. However, some state health department interviewees characterised the HP policy and practice environment as the “*glass is half full*” because of the implementation of the SA Public Health Act, which laid out governance structures for collaboration between state and local governments.

### Dominant themes and feedback mechanisms

**Fig. 2:**
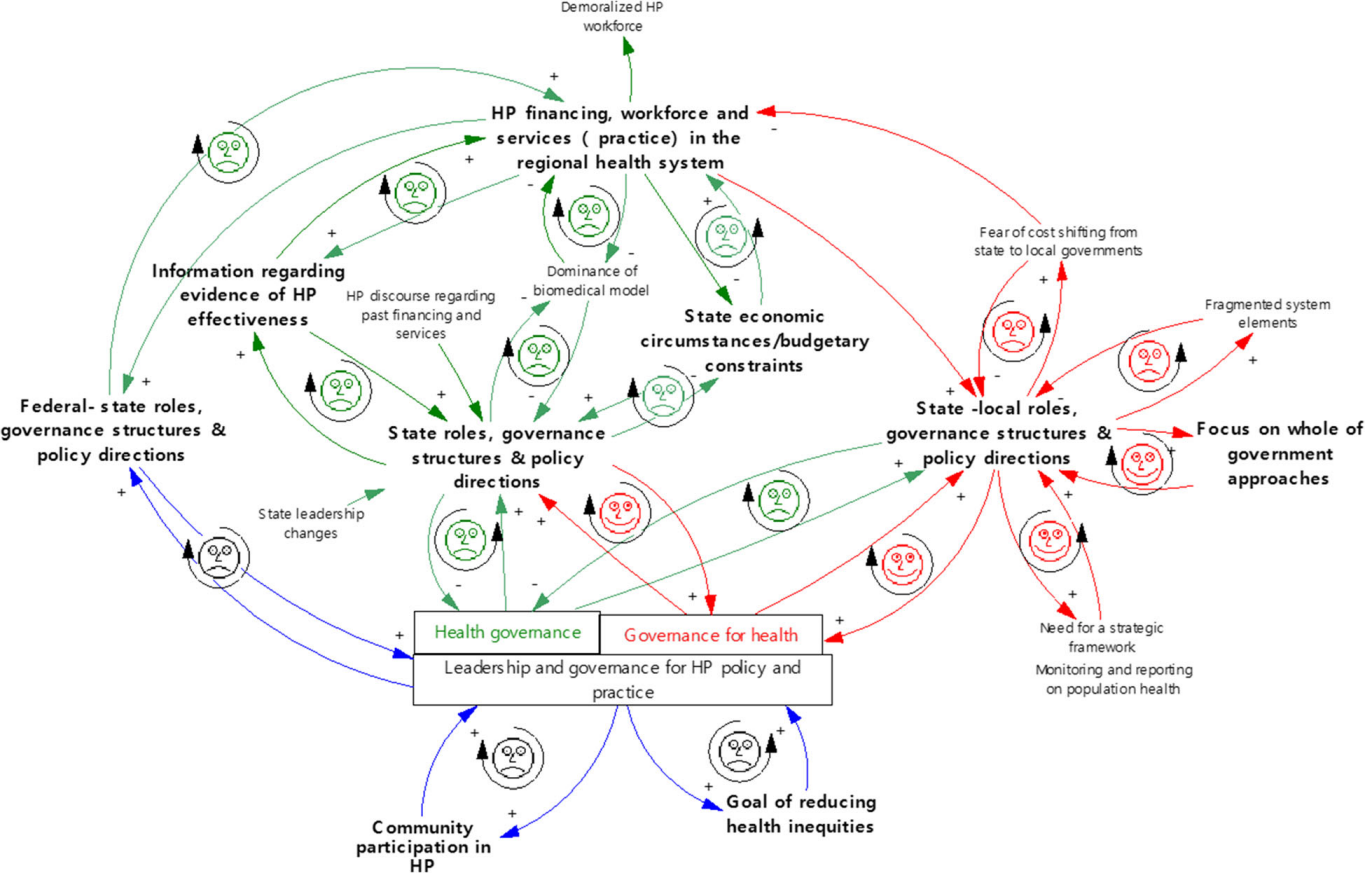
Causal loop diagram

Table 5 provides a list of key findings and illustrates, through check marks, if they were found in document review and/or interview data. Dominant themes are those where key findings were found in both document review and interview data (two check marks). In the following section, dominant themes are reported and feedback mechanisms identified. All feedback mechanisms are illustrated in one causal loop diagram (Fig. 2) and dominant themes are indicated through bold font. A detailed explanation of each feedback mechanism can be found in Additional file 1 (Description of causal links and feedback mechanisms).

#### Goal of reducing health inequities

There was little support for or discussion of reducing health inequities in either the document review or the interview data. When reducing health inequities was discussed (NGO/Intersectoral Network interviewee; SA Health group interview) it was limited to addressing the needs of disadvantaged people and discussion did not address the social gradient in health [4]. The lack of strong leadership and governance by way of strategic policy frameworks for the goal of reducing health inequities forms a detrimental feedback loop or a vicious cycle that inhibited HP policy and practice (Fig. 2).

#### Community participation in HP

Although many documents identified and supported community participation in HP (2003–2013), the Review and SA Health’s Response greatly diminished this because of cuts to the financing, workforce and services. The SA Public Health Act included a participation principle; however, it is weak in comparison to empowerment approaches to strengthen community action [2, 54]. Although community participation was discussed by several interviewees as being important (particularly in working with disadvantaged populations), it was reported to be “*old hat*”, “*not modern*” (#2/NGO/Health service). Interviewees reported a retreat from the strong history of using community development approaches in primary healthcare services in the case health system (#29 and #50/NGO/Intersectoral Network). The lack of leadership and policy directions for HP policy and practice to facilitate community participation also formed an inhibiting and vicious feedback mechanism (Fig. 2).

#### Federal-state-local roles, governance structures and policy directions

The three levels of government figured prominently in the data. The Review and SA Health’s Response altered governance structures significantly as HP leadership at the local and regional (case) health system level was conceded to (1) the federal government through the federally funded and regionally managed Medicare Local and to (2) local governments through the SA Public Health Act. The Review was frequently discussed in interviews with stakeholders from all sectors. Stakeholders reported that the lack of implementation of an original federal-state National Health Care Reform Agreement [55] was a key factor and, as a result, HP was a “*casualty*” of the politics between levels of government because no level of government accepted leadership responsibilities. One interviewee was particularly expressive with respect to this: “*what we’ve got is an ad hoc, politically influenced, double–dipping, cherry picking, State-Commonwealth split*” (#2/NGO/Health Service).

##### Federal-state level

The lack of delineation of federal-state roles, governance structures and policy direction played out with respect to the state cuts to HP financing, workforce and services in the case health system because of a false assumption that the federal government’s Medical Local initiative would be doing this work. Thus, inhibiting and vicious feedback loops were found (Fig. 2).

##### State level

Most documents discussed the important leadership and health governance role of the state government in reorienting health services toward HP to some extent. Examples include discussion of moving from “*an illness focused to a health focused system*” ([56], p. 14) and enhancing HP through primary healthcare services [57]. The most striking finding was the abdication of these functions in 2013 following cuts to HP recommended in the Review and SA Health’s Response. The perceived lack of information regarding HP effectiveness was one reason given for cuts to HP financing, workforce and services. Almost all stakeholders, except some from SA Health, discussed the Review’s perspective on the effectiveness of HP. Many interviewees shared the concern that the Review did not use an appropriate evaluation framework based upon HP principles and practices. Although many stakeholders reported that evaluating the effectiveness of HP was a great challenge, several suggested that expecting to generate evidence of effectiveness in the case health system was futile given that so little had been invested in HP initiatives. There were three vicious and inhibiting feedback mechanisms with respect to the lack of state leadership and health governance, the lack of information regarding evidence of HP effectiveness, and cuts to HP financing, workforce and services (Fig. 2).

Furthermore, the Review and SA Health’s Response identified the state’s economic circumstances and budgetary constraints due to rising healthcare expenditures as key factors influencing cuts to HP financing, workforce and services. Calls in earlier documents for strong leadership and health governance to ensure adequate and sustained funding for HP were unheeded. Stakeholders used phrases such as “*soft target*” and “*easy target*” to explain the HP cuts (e.g., #4/Local Council, #35/NGO/Professional Association). One interviewee voiced what others reported, namely that the primary concern of the newly appointed Minister of Health was “*the great hole in the Health budget*” and cuts to HP were a “*quick political win*” in an election year (#2/NGO/Health service). Others reported that the cuts were very abrupt and “*they’re cutting their nose off to spite their face because of their focus on a balanced budget*” (#9/Medicare Local) and “*some things seem to pass with little controversy like enormous new ovals* [cricket stadiums] *while small amount of money are cut*” (#46/NGO/Professional Association). In sum, stakeholders saw the cuts to HP financing, workforce and services to be part of an austerity agenda to put reducing budget deficits above HP policy and practice. One feedback mechanism links state roles, governance structures and policy directions with state economic circumstances and budgetary constraints and another links the latter with cuts to HP financing, workforce and services (Fig. 2). These are both inhibiting feedback loops that act to balance or stabilise the system to an undesirable state. That is, the feedback loops illustrate how healthcare costs are constrained through cuts to HP financing, workforce and services.

##### State-local level

State policy directions resulting from the Review, SA Health’s Response and the SA Public Health Act emphasised leadership and health governance for HP at the local or regional levels (local governments and the Medicare Local in the case health system). Cuts to HP financing, workforce and services in the Local Health Network were unveiled alongside a redirection of resources to chronic disease management. Many interviewees reported being demoralised because of HP’s decline. Further, interviewees commonly discussed the consequences of a policy that implied cost shifting from state to local governments for HP with no new HP initiatives being planned. For example, one interviewee reported: “*I see a lot of cost and expenses so no one is looking to really take it* [HP] *on board because they know it’s like a poisoned chalice*” (#4/Local Council). Medicare Local interviewees pointed out that they mostly worked from a biomedical or clinical model and had no dedicated funding or workforce for HP. From this, an inhibiting feedback loop was identified (Fig. 2).

Conversely, the policy context was somewhat favourable for state and local leadership in governance for health (Table 4) through developing partnerships and intersectoral collaboration. All documents discussed partnerships and intersectoral collaboration to some extent and the SA Public Health Act offered clear policy directions for partnership development between the state government departments, local government and other organisations. Furthermore, there is a historical richness in SA documents (2003–2013) regarding policy direction for governance for health, particularly whole-of-government or Health in All Policies approaches. For example, the Adelaide Statement on Health in All Policies [58] emphasises the need for new governance structures and processes for partnerships in order to join up efforts to improve population health. The intent to build healthy social, economic and environmental policies underlying this document carried forward to the SA Public Health Act.

Stakeholders from all sectors reported that the SA Public Health Act was the key policy driver for HP in 2013. While it provided state and local support for leadership and governance for health, sectors and system elements were reported to be fragmented and the structures and processes for partnership development and collaboration were in their infancy. There was knowledge and a certain pride among many interviewees that the whole-of-government approach was in play within the state government; however, local governments appeared to have minimal involvement in the case health system. Building healthy public policy was explained by SA Health interviewees in terms of the SA Public Health Act being “*a real drive for Health in All Policies*” at the state and local government levels. Figure 2 illustrates the relationship between governance for health, state-local roles and whole-of-government approaches as facilitating feedback loops or virtuous cycles that are favourable for HP.

## Discussion

Our use of a causal loop diagram enabled us to identify the complex interplay of factors that affect HP and explain why the case study health system no longer supported HP. We found a complex picture with numerous interactions and feedback mechanisms represented in the causal loop diagram. The approach used helped us understand the patterns in system behaviour. Doing this makes it possible to identify potential opportunities to disrupt or slow down vicious feedback mechanisms and/or amplify those that are virtuous cycles. The majority of feedback loops in the causal loop diagram were vicious cycles that would need to be disrupted or changed for HP to thrive in the case study heath system. Changing even one feedback loop could change the emergent order of the system because system behaviour is a product of how the parts fit together and not how they act separately. Thus, feedback mechanisms can be seen as leverage points to strengthen systems [59] and this section highlights potential implications and links to other literature.

### Disrupt vicious feedback mechanisms that inhibit HP

Improving HP policy and practice requires changing the feedback loop that inhibited the system goal of reducing health inequities. Strong leadership and governance could ensure that strategic policy frameworks targeting health inequities exist and facilitate policy coherence between levels of government [60] to address populations experiencing disadvantage, closing the gap in inequities and flattening the social gradient [61]. This concurs with Kickbusch and Gleicher’s view that “*the actions needed to improve health and reduce health inequities require new systems-based governance and delivery mechanisms that take account of interdependencies, complexity and the need for whole-of-government and whole-of-society co-production of population health*” ([62], p. 19).

Similarly, changing the inhibiting feedback loop with respect to community participation in HP is a notable opportunity. Active community participation is essential to effective HP policy and practice [2, 6, 7, 63–65]. A virtuous cycle to encourage this could be established through strong policy statements that embed community participation in all HP planning, implementation and evaluation.

A feedback mechanism illustrates the leadership and governance challenges between the federal and state governments. This feedback mechanism is an important leverage point and needs to be disrupted, yet actions are highly political. As Bennett [66] notes, there is an ongoing blame game between the federal and state levels of government in Australia and this feedback mechanism may prove hard to change unless a window of opportunity develops where both federal and state governments have a strong desire to improve HP practice.

At the state level, the feedback mechanisms that illustrate the dominance of budgetary constraints is clearly a challenge to HP given the resultant cuts to financing and workforce. Without disrupting these feedback loops, a void in HP policy and practice will remain. These feedback loops point to the vulnerability of HP financing and lend support to calls for political will and leadership and governance structures to leverage dedicated funding for HP in Australia [67]. Duckett and Willcox state that “*health expenditure and health financing policies are rarely off the policy agenda*” ([68], p. 42). They further report that health expenditures in Australia are “*what would be expected given its GDP*” ([68], p. 42), opening debate about assertions such as those in the Review. It is beyond the scope of this paper to enter into debate; however, it appears illogical for policy-makers to target HP when public health as a whole in Australia represents only 2% of all health expenditures and could lead to savings in healthcare [68, 69].

There are other feedback mechanisms at the state level that require change in order to strengthen HP. Addressing the vicious feedback mechanism associated with the lack of evidence of HP effectiveness will require leadership and health governance to allocate sufficient resources to implement and evaluate sustained and promising HP actions and strategies [25, 70]. Importantly, a systems approach focused on addressing the broad political and structural determinants of health is needed [3]. Rutter et al. [1] state that a complex system model of evidence is necessary: “*Although it is important for public health policy to be guided by evidence, if this evidence predominantly supports individual-level interventions that have minimal reach and effect across populations, the benefits of being informed by the existing evidence base might be illusory*”. Beyond this, the abdication of leadership and health governance for HP did nothing to address this challenge and opposed calls for health systems to address the paucity of intervention research [71].

Turning to the state-local level, the inhibiting feedback loop that links the lack of leadership and health governance for reorienting health services to HP produced a policy vacuum. In other words, without disrupting the feedback loop, system elements, such as local governments and the Medicare Local, will stabilise around the policy vacuum and nothing will change in HP services because of the lack of financing and workforce building blocks. International documents have called for the health sector to reorient health services and lead HP since at least the Declaration of Alma Ata [6] in 1978 and, more recently, in the Rio Political Declaration on Social Determinants of Health [8] in 2011. However, this has been a long-standing challenge primarily because of entrenched factors including powerful vested interests and the dominance of the biomedical model [10, 72].

### Amplify virtuous feedback mechanisms that facilitate HP

Virtuous cycles were identified with respect to governance for health through partnerships and intersectoral collaboration and the key implication is the need to amplify these cycles. Leadership and governance for health through partnership development and intersectoral collaboration is critically important to HP policy and practice because of the complex interactions between factors that contribute to population health that are beyond the influence of any one sector in society [4, 6, 73, 74]. Amplification of these feedback loops would strengthen the implementation of the SA Public Health Act and whole-of-government or Health in All Policies approach at both state and local government levels. Whole-of-government approaches to HP have been called for many years [2, 75] and this research identifies great opportunity to build upon the rich history in SA. Legislation can be a powerful driver for collaboration and the SA Public Health Act provides a platform for aligning policies at state and local government levels simultaneously. There is a note of caution, however, as the lack of health governance for HP in reorienting health services, as discussed above, has the potential to have a negative impact upon governance for health [76]. That is, if the state and federal government do not champion HP within their respective health sectors, then why would other sectors and partners champion HP?

### Limitations

When applying complex systems approaches it is necessary to define what is within the boundary of the system and what is out. This inevitably means that elements important to the system may be defined as outside of it [77]. In this study, inclusion of stakeholders from sectors and system elements, such as social service agencies and schools, might have offered different and useful perspectives.

The WHO framework was a useful foundation to study the case health system. However, the adapted definitions of the building blocks for a multisectoral health system for HP, being novel, would benefit from further applications and testing with policy-makers and practitioners to assess their value.

Creating causal loop diagrams in conjunction with group model building processes with stakeholders is called for in the literature [78]. Time and resource constraints did not permit this step. Although the research team undertook extensive discussion and achieved consensus on the causal loop diagram, facilitating a group model building process would have been preferable to not only gain their perspectives but to engage in discussion about implications, priority leverage points and actions to strengthen HP in the case health system. Thus, future research could build upon this research and use participatory systemic inquiry methods [79].

## Conclusion

Leadership and governance for HP were found to be central factors that influenced HP policy and practice confirming findings from other jurisdictions around the world [62]. This study demonstrates its critical importance and adds urgency to the need for increased and strong advocacy for HP. The application of a complex systems approach to HP policy and practice addressed a gap in the literature. Our new methods have made visible the complex web of factors that influenced HP in an Australian multisectoral health system. Our approach was pioneering in that we combined health system building blocks and feedback mechanisms as leverage points [59]. Our causal loop diagram offered a picture of the broad array of interdependent facilitating and inhibiting factors that can be targeted to improve HP policy and practice.

## Additional file

Additional file 1: Descriptions of causal links and feedback mechanisms. (DOCX 21 kb)
