# Supplementary material for: The value of a causal loop diagram in exploring the complex interplay of factors that influence health promotion in a multisectoral health system in Australia
Source: Health Res Policy Syst. 2018 Dec 29;16:126. doi: 10.1186/s12961-018-0394-x (PMC6310960; doi:10.1186/s12961-018-0394-x)
Supplement: Supplementary file 1 — Descriptions of causal links and feedback mechanisms. (DOCX 21 kb) [file 12961_2018_394_MOESM1_ESM.docx]

**Additional file 1. Descriptions of causal links and feedback mechanisms**

Table 1. Leadership and governance and feedback loops regarding health inequities and community participation in.

| **Findings** | **Document review** | **Stakeholder interviews** | **Leadership and governance** | |
| --- | --- | --- | --- | --- |
|  |  |  | Leadership/ health governance | Leadership/  governance for health |
| Lack of strong support for/discussion of reducing health inequities | 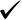 | 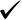 | 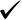 | 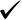 |
| Lack of support for ensuring community participation in HP | 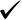 | 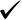 | 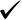 | 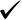 |
| **Description of causal links and feedback loops** | | | | |
| Lack of strong support for/discussion of **reducing health inequities** negatively influences leadership and governance for HP policy and practice (+); lack of leadership and governance for HP policy and practice negatively influences support for/discussion of reducing health inequities (+). | | | Positive feedback loop that inhibits HP (vicious cycle) | |
| Lack of support for **community participation** in HP negatively influences leadership and governance for HP policy and practice (+); lack of leadership and governance for HP policy and practice negatively influences support for community participation in HP (+). | | | Positive feedback loop that inhibits HP (vicious cycle) | |

Table 2. Leadership and governance and numerous feedback loops regarding federal-state-local government roles, governance structures and policy directions.

| **Findings** | **Document review** | **Stakeholder interviews** | **Leadership and governance** | |
| --- | --- | --- | --- | --- |
|  |  |  | Leadership/ health governance | Leadership/  governance for health |
| **Federal–state-local roles, governance structures and policy directions** [Calls for enhanced intergovernmental relations (federal-state-local); delineation of leadership roles and governance structures; impact of federal and state policy directions] | 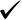 | 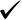 | 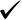 | 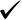 |
| **Description of causal links and feedback loops** | | | | |
| Lack of **federal-state roles**, governance structures and policy directions negatively influenced leadership and governance (+) for HP; lack of leadership and governance for HP lead to the lack of clear federal state roles, governance structures and policy directions (+).  Lack of **federal-state roles**, governance structures and policy directions negatively influenced HP financing, workforce, and services (practice) in the regional health system (+); the lack of HP financing, workforce, and services (practice) in the regional health system further lead to the lack of **federal-state roles,** governance structures and policy directions. | | | Positive feedback loop that inhibits HP  (vicious cycle)  Positive feedback loop that inhibited HP (vicious cycle) | |
| **State roles**, governance structures and policy directions diminished leadership and health governance (-); lack of leadership and health governance diminished state roles, governance structures and policy directions for HP (+). | | | Negative feedback loop that inhibits HP (balancing cycle) | |
| **State roles,** governance structures and policy directions positively influenced leadership and *governance for health* (+); leadership and governance for health at the state level positively influenced state roles, governance structures and policy directions (+). | | | Positive feedback loop that facilitates HP (virtuous cycle) | |
| **State-local roles**, governance structures and policy directions diminished leadership and health governance (-); diminished leadership and health governance diminished *State-local roles*, governance structures and policy directions (+). | | | Negative feedback loop that inhibits HP (balancing cycle) | |
| **State-local roles**, governance structures and policy directions positively influenced leadership and governance for health (+); leadership and governance for health positively influenced state-local roles, governance structures and policy directions (+). | | | Positive feedback loop that facilitates HP (virtuous cycle) | |

Table 3. Leadership and health governance and feedback loops with respect to state roles, governance structures and policy direction; evidence of HP effectiveness; state economic circumstance/budgetary constraints; and the dominance of the biomedical model.

| **Findings** | **Document review** | **Stakeholder interviews** | **Leadership and governance** | |
| --- | --- | --- | --- | --- |
|  |  |  | Leadership/ health governance | Leadership/ governance for health |
| Lack of information/evidence of HP effectiveness | 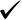 | 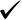 | 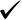 |  |
| Impact of state economic circumstances/budgetary constraints | 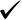 | 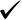 | 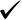 |  |
| Dominance of biomedical model |  | 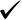 | 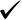 |  |
| **Impact of state leadership changes* |  | 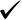 | 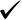 |  |
| **Impact of HP discourse regarding past financing and services* |  | 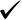 | 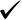 |  |
| **Demoralisation of HP workforce* |  | 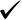 | 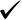 |  |
| **Description of causal links and feedback loops** | | | | |
| Lack of **information/evidence of HP effectiveness** negatively influenced the **state roles,** governance structures and policy directions (+) for reorienting health services toward HP (+); state roles, governance structures and policy directions in health governance reorienting health services diminished information regarding evidence of HP effectiveness (+). | | | Positive feedback loop that inhibited HP (vicious cycle) | |
| Poor state economic circumstances/budgetary constraints negatively influenced **state roles**, governance structures and policy directions for HP (+); **state roles**, governance structures and policy directions for HP positively influenced state economic circumstances/budgetary constraints (-). | | | Negative feedback loop that inhibited HP (balancing cycle) | |
| Dominance of biomedical model negatively influenced **state roles**, governance structures and policy directions for HP (-); state-local roles, governance structures and policy directions for HP were negatively influenced by the dominance of biomedical model (-). | | | Positive feedback loop that inhibited HP (vicious cycle) | |
| **The impact of state leadership changes and the HP discourse regarding past financing and services were seen to influence* ***state-local roles*** *and governance structures. However, there was no data to support this being a causal link. The demoralisation of HP workforce was seen to be a result of cuts to HP financing, workforce, and services (practice) in the regional health system, however this was not seen to be a reciprocal relationship* | | | | |

Table 4. Leadership and governance and feedback loops regarding HP financing, workforce and services (practice).

| **Findings** | **Document review** | **Stakeholder interviews** | **Leadership and governance** | |
| --- | --- | --- | --- | --- |
|  |  |  | Leadership/ health governance | *Leadership/ governance for health |
| Cuts to/lack of HP financing | 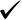 | 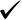 | 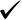 | 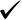 |
| Cuts to/the need for HP workforce capacity | 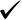 | 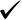 | 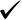 | 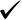 |
| Cuts to/limited HP services (practice) | 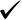 | 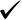 | 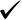 | 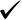 |
| **Description of causal links and feedback loops** | | | | |
| Dominance of the biomedical model negatively influenced **HP financing, workforce and services (practice**) in the regional health system (-); HP financing, workforce and services (practice) in the regional health system positively influenced the dominance of the biomedical model (-). | | | Positive feedback loop that inhibited HP (vicious cycle) | |
| Lack **of information/evidence of HP effectiveness** negatively influenced **HP financing, workforce and services (practice)** (+); the lack of HP financing, workforce and services (practice) negatively influenced the lack of information/evidence of HP effectiveness (+). | | | Positive feedback loop that inhibited HP (vicious cycle) | |
| Poor **state economic circumstances**/budgetary constraints negatively influenced cuts to **HP financing, workforce and services (practice)** (+); cuts to HP financing, workforce and services (practice) in the regional health system positively influenced the poor state economic circumstances/budgetary constraints (-). | | | Negative feedback loop that inhibited HP (balancing cycle) | |
| **See Table 8 for links to leadership and governance for health.* | | | | |

Table 5. Leadership and governance for health and feedback loops regarding cost shifting, fragmentation, whole-of-government approaches, strategic frameworks, and monitoring and reporting on population health.

| **Findings** | **Document review** | **Stakeholder interviews** | **Leadership and governance** | | |
| --- | --- | --- | --- | --- | --- |
|  |  |  | Leadership/ health governance | | Leadership/ governance for health |
| Fear of cost shifting from state to local governments |  | 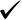 |  | | 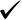 |
| Fragmented system elements |  | 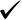 |  | | 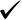 |
| Calls for/focus on whole-of-government approaches | 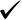 | 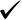 |  | | 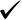 |
| Need for a strategic framework |  | 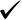 |  | | 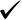 |
| Support for monitoring and reporting on population health | 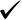 |  |  | | 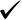 |
| **Description of causal links and feedback loops** | | | | | |
| **State-local government roles**, governance structures and policy directions heightened fears of cost shifting from the state to local governments (+): heightened fears of cost shifting from the state to local governments negatively influenced state-local government roles, governance structures and policy directions (-). | | | | Negative feedback loop that inhibited HP (balancing cycle) | |
| Heightened fears of cost shifting from the state to local governments negatively influenced **HP financing, workforce and services (practice)** (-); lack of HP financing, workforce and services (practice) negatively impacted state-local government roles, governance structures and policy directions (+). | | | | Negative feedback loop that inhibited HP (balancing cycle) | |
| Fragmented system elements negatively influenced state-local government roles, governance structures and policy directions (lack of collaborative mechanisms) (-); state-local government roles, governance structures and policy directions are negatively influenced by fragmented system elements and the lack of collaborative mechanisms (-). | | | | Negative feedback loop that inhibited HP (balancing cycle) | |
| **Focus on whole-of-government approaches** positively influenced **state-local government roles (+)**; state-local government roles, governance structures and policy directions was positively influenced by focus on whole-of-government approaches (+). | | | | Positive feedback loop that facilitates HP (virtuous cycle) | |
| Need for a strategic framework and support for monitoring and reporting on population health were positively linked to s**tate-local government roles**, governance structures and policy directions (+); state-local government roles, governance structures and policy directions were positively linked to the need for a strategic framework and supported monitoring and reporting on population health (+). | | | | Positive feedback loop that facilitates HP (virtuous cycle) | |
